# The Effect of Abiotic Stresses on the Protein Composition of Four Hungarian Wheat Varieties

**DOI:** 10.3390/plants11010001

**Published:** 2021-12-21

**Authors:** Dalma Nagy-Réder, Zsófia Birinyi, Marianna Rakszegi, Ferenc Békés, Gyöngyvér Gell

**Affiliations:** 1 Department of Biological Resources, Agricultural Institute, Centre for Agricultural Research, ELKH, H-2462 Martonvásár, Hungary; reder.dalma@atk.hu (D.N.-R.); birinyi.zsofia@atk.hu (Z.B.); 2 Doctoral School of Biology, Institute of Biology, ELTE Eötvös Loránd University, H-1117 Budapest, Hungary; 3 Cereal Breeding Department, Agricultural Institute, Centre for Agricultural Research, ELKH, H-2462 Martonvásár, Hungary; rakszegi.mariann@atk.hu; 4 FBFD PTY LTD., Hull Road 34, Beecroft, Sydney, NSW 2119, Australia; firinc47@gmail.com

**Keywords:** wheat, climate change, abiotic stress, HPLC, ELISA, heat-and drought stress, protein composition

## Abstract

Global climate change in recent years has resulted in extreme heat and drought events that significantly influence crop production and endanger food security. Such abiotic stress during the growing season has a negative effect on yield as well as on the functional properties of wheat grain protein content and composition. This reduces the value of grain, as these factors significantly reduce end-use quality. In this study, four Hungarian bread wheat cultivars (*Triticum aestivum* ssp. *aestivum*) with different drought and heat tolerance were examined. Changes in the size- and hydrophobicity-based distribution of the total proteins of the samples have been monitored by SE- and RP-HPLC, respectively, together with parallel investigations of changes in the amounts of the R5 and G12 antibodies related to celiac disease immunoreactive peptides. Significant difference in yield, protein content and composition have been observed in each cultivar, altering the amounts of CD-related gliadin, as well as the protein parameters directly related to techno-functional properties (Glu/Gli ratio, UPP%). The extent of changes largely depended on the timing of the abiotic stress. The severity of the negative effect depended on the growth stage in which abiotic stress occurred.

## 1. Introduction

Extreme environmental events in recent years, and the significant increase in global temperatures and climatic variability, drew attention to agricultural crop productivity, quality and plant resistance to extreme environmental conditions and food security.

Wheat is a very important crop, which provides raw material for staple food diets. Maintaining grain quality as well as yield under extreme climatic conditions is crucial for human nutrition and end-use functional properties. Wheat grain storage proteins represent 70–80% of the protein content, depending on species [1]. They are key players in wheat-related diseases, like the autoimmune reaction of celiac patients and wheat allergy sufferers. Gluten proteins consist of two major fractions: monomeric gliadins and polymeric glutenins [2]; their contribution to dough properties has long been recognized [3]. They form a typical protein profile in modern wheat genotypes, which confers viscosity and elasticity properties to the dough.

Dough strength is an important bread-making quality of wheat flour and plays a key role in the food industry. Nutritional studies contribute to understanding dough rheological characteristics of wheat flour. They can be characterized both by the quality and quantity of total gluten, as well as by the composition and quantity of gluten polypeptides. Different wheat cultivars show differences in gluten strength determined by storage protein composition, in the composition and the total amount of HMW-glutenin subunits, the glutenin-to gliadin ratio, and the relationship between SDS-soluble and SDS-insoluble protein polymers [4].

With the rapidly changing climate, combined abiotic stress factors—lack of water and elevated temperatures—occur more often. Previous studies revealed that dough properties, hence baking-quality, highly depend on environmental factors. It was shown that the significant traits of wheat yield and grain quality occur mainly during the grain filling stage [5]. Drought stress is one of the major environmental constricting factors to plant growth and productivity. Water shortage during growing season leads to an increase in the relative protein content and the ratio of high molecular weight (HMW) to low molecular weight (LMW) glutenin subunits, while also a decrease in grain yield [6]. Drought stress causes a rise in the relative protein content, and also elevates the reduction and deterioration of grain quality.

Besides the individual stress effects in a few studies, drought and heat combined stress were also examined with regard to grain composition. Combined effects of extreme weather circumstances, such as high temperature with drought, are expected to be the main yield decreasing factors [7,8]. Sensitivity to abiotic stress factors highly depends on the genotypes, the developmental stages of the plants, and the severity and intensity of the stress. Heat and drought stress occurring at the reproductive stage may cause serious effects on crop yields [9].

Zhao et al. [10] also found evidence that the protein composition is sensitive to drought during grain filling, resulting in deterioration in dough quality due to a reduction in the glutenin-to-gliadin ratio and the percentage of very large glutenin polymers.

Proteomic analysis provides an effective tool to monitor changes in the wheat proteome, triggered by different environmental stress effects such as drought and heat, at the genome-wide level [11,12]. These abiotic factors generate complex proteomic changes in wheat grains and influence growth, biochemical pathways, and physiological processes. The ability for adaptation and/or tolerance of wheat cultivars to abiotic stress is certainly a crucial selection criterion in wheat breeding.

In our study, the effects of individual and combined drought and heat stress—characteristic to the Carpathian Basin—were examined using a climate prediction model on Hungarian bread wheat cultivars. The main purpose of the research was to monitor the changes in grain storage protein composition, of pre-selected heat-sensitive, drought-sensitive, heat-tolerant, and drought-tolerant Hungarian bread wheat cultivars, in response to abiotic stress factors occurring at anthesis and post-anthesis periods. Phytotron climate chamber experiments were performed to evaluate temperature and drought, and their combined abiotic stress effects, and to examine their impact on the wheat grain storage protein composition and celiac-related epitope levels.

## 2. Results and Discussion

The number of extreme climate-related events shows an upward trend due to global climate change, and the negative effects of abiotic stresses will occur in the Carpathian Basin which has a continental-type climate [13]. Our study focuses on the comparison of abiotic stress resistance of different bread wheat varieties. The long-term goal is to find appropriate varieties that are of good quality and have good yield, under the extreme local climatic conditions, and use them as breeding lines.

Examination of different wheat varieties in view of end-product quality is very basic in wheat breeding and the food industry. End-product quality includes both techno-functional properties such as gluten strength, nutritive attributes, and properties related to health. In the case of bread-making, the characterisation of gluten protein composition is the most important factor, while for other products such as noodles, the investigation of starch properties plays an essential role. Qualitative and quantitative characterisation of gluten proteins determining the glutenin-to-gliadin ratio, unextractable polymeric protein percentage (UPP%), as well as information about the glutenin allelic composition, shed light on dough strength and extensibility—the two key factors determining bread-making quality [14]—while knowledge of starch characteristics determinants of viscosity parameters is important in noodle production. The characterization of nutritive and health-related attributes require information on protein composition—the amounts of harmful gluten- and non-gluten proteins (certain gliadin and LMW glutenin polypeptides containing coeliac and allergen epitopes, amylase trypsin inhibitors (ATIs), and the amounts of several non-starchy carbohydrates such as fructans, arabinoxylan, and beta-glucan. In the case of bread wheat, several studies have confirmed that considerable changes occur in the expression level of different storage protein fractions and non-starchy carbohydrate components [15]. These changes can be accurately monitored under controlled phytotron conditions, and the results can be used to pre-model the climate change-induced qualitative changes and their impact on celiac-related proteins

In this study, an old Hungarian cultivar (Bánkúti 1201) and three bread wheat varieties bred in Martonvásár, Hungary (Mv Mambó, Mv Palotás, and Mv Hombár) were examined. Based on a previous glasshouse study, four genetically different varieties—differing in their abiotic stress resistance—were selected for detailed analyses of protein composition from the aspect of climate adaptation behavior. Our results showed that the phenological stage at which the abiotic stress was applied severely affected the alteration of the quantitative and qualitative parameters. All physiological and chemical parameters of the selected cultivars grown under different abiotic stress conditions are interpreted and discussed at two levels:

- Analytical data are compressed in [% of the whole meal sample] dimension, by combining raw protein content size exclusion- and reverse-phase high performance liquid chromatography (SE- and RP-HPLC) data as described in the Method section.
- Alterations caused by the abiotic treatments are illustrated by comparing the analytical data to the percentage of the corresponding control data.

### 2.1. Effect of Abiotic Stress Treatments on the Physiological Parameters and Yield

Four Hungarian bread wheat cultivars, including a drought-sensitive (Bánkúti 1201), a drought-tolerant (Mv Mambó), a heat-sensitive (Mv Palotás), and a heat-tolerant (Mv Hombár) variety, were studied in the experiment. The alteration of the physiological parameters of the selected samples caused by the different abiotic treatments is shown in Supplementary Table S1.

Based on our previous study the effect of the high-temperature stress depends a lot on the timing the treatment is applied. Thus, before flowering, the formation and the number of spikelets are most impacted by the temperature stress, while at grain filling stage, the floral development and the number of the grains are most affected [16]. Significant effect of the elevated temperature stress on the grain development was examined in several studies and the results showed shorter maturation times and increased storage protein accumulation [17,18].

#### 2.1.1. Drought Stress

In our study, drought stress treatment at anthesis (***DS_anth***) led to a significant yield loss of the examined cultivars (between 17.94–85.08% compared to the control), while in the case of the drought-tolerant Mv Mambó, a 22.03% improvement was observed. The lack of water at the grain filling stage (***DS_postanth***) caused 51.82% and 30.14% yield loss of drought-sensitive and heat-tolerant cultivars, but generated a yield increase in drought-tolerant and heat-sensitive cultivars of 7.99 and 14.87% compared to the control. Due to ***DS_anth*** treatment, the thousand kernel weight was lower than the control by 4.7–61.93%, except for the drought-tolerant Mv Mambó. Compared to the control, ***DS_postanth*** caused less reduced thousand kernel weight in drought-sensitive and heat-tolerant cultivars (by 43.71 and 5.05%), but increased the values of the drought-tolerant and heat-sensitive cultivars by 19.21 and 18.36%, respectively. Yield data are summarised in Supplementary Table S1.

#### 2.1.2. Heat Stress

The high temperature at anthesis (***HT_anth***) improved the crop yield by 15.56–167.94%, and heat stress occurring at grain filling stage (***HT_postanth***) caused a 13.46–119.78% increase. However, in the case of the drought-sensitive cultivar, Bánkúti 1201, the reduction observed was caused by ***HT_postanth***.

A similar tendency occurred in the case of the thousand kernel weight (TKW) as well. ***HT_anth*** generated a 35.94–89.17% increases in TKW (except for the drought-sensitive Bánkúti 1201) and a 0.49–40.02% increase at 6–10 days post-anthesis.

#### 2.1.3. Combined Stress

Combined temperature and drought stress at grain filling stage (***CMB_postanth***) caused significant degradation in the yield (by 48.03–78.35% compared to the control) and the thousand kernel weight (by 30.01–54.05% compared to the control), but resulted in even more significant yield loss (77.97–100%) at anthesis.

Analysis of variance carried out on the physiological data (Table 1) indicated that, except for the number of spikelets per main spike, significant differences exist among the extent of alteration of parameters caused by the different abiotic treatment for all four cultivars. Comparison of the F values reveals that the combined early stress produced the most significant abiotic effect on each parameter. The drop in yield caused by the combined abiotic stress treatment resulted in such a severe yield decrease that pre-planned analyses of the protein composition in these samples could not be performed.

### 2.2. Effect of Abiotic Stress on Protein Content and Protein Composition

The effects of environmental stress factors frequently manifest in the rebalance of gluten protein accumulation, altering gluten protein composition. These alterations consequently lead to elevation of grain protein content and drop of the end-use quality under stress conditions. In our study investigating four varieties with different abiotic stress resistance characteristics, strong significant alterations have been observed in the physiological and chemical parameters regarding the effect of the applied drought and heat stresses. We were looking for the connection between the alteration in certain amounts (e.g., UPP%, glutenin-to-gliadin ratio) and abiotic stress effects at anthesis and grain filling stages.

**Figure 1:**
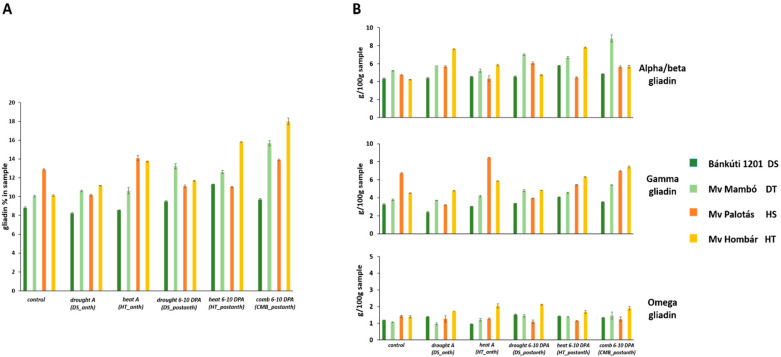
Gliadin content (**A**) and composition (**B**) of samples altered by different abiotic treatments. Bk—Bánkúti 1201; M—Mv Mambó; P—Mv Palotás; H—Mv Hombár. *DS_anth*: drought stress at anthesis; *HT_anth*: high temperature at anthesis; *CMB_anth*: combined stress at anthesis; *DS_postanth*: drought stress at post-anthesis; *HT_postanth*: high temperature at post-anthesis; *CMB_postanth*: combined stress at post-anthesis.

**Figure 2:**
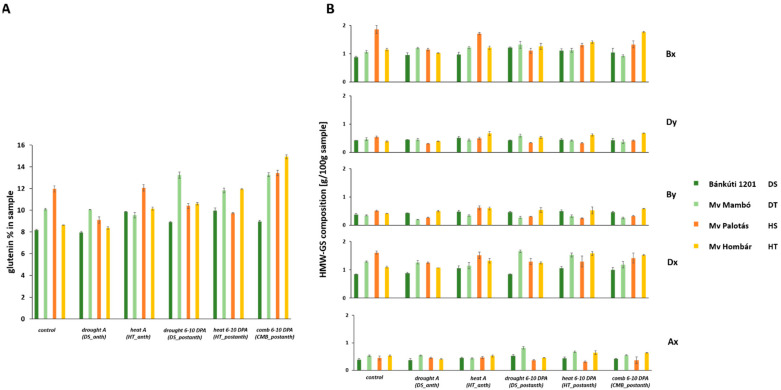
Glutenin content and high molecular weight glutenin subunits (HMW GS) composition of samples altered by different abiotic treatments. (**A**) Glutenin content [% of the sample] was determined from data derived from the SE-HPLC separation of the total protein content multiplied by the protein content of the sample. (**B**) The individual amounts of 5 (Ax, Bx, By, Dx and Dy) HMW GS subunits [% of the sample] were calculated from the data derived from the RP-HPLC separation of glutenin proteins, multiplied by the glutenin content of the total protein, determined by the SE-HPLC separation and multiplied by the protein content of the samples. (Bk—Bánkúti 1201; M—Mv Mambó; P—Mv Palotás; H—Mv Hombár). *DS_anth*: drought stress at anthesis; *HT_anth*: high temperature at anthesis; *CMB_anth*: combined stress at anthesis; *DS_postanth*: drought stress at post-anthesis; *HT_postanth*: high temperature at post-anthesis; *CMB_postanth*: combined stress at post-anthesis.

**Figure 3:**
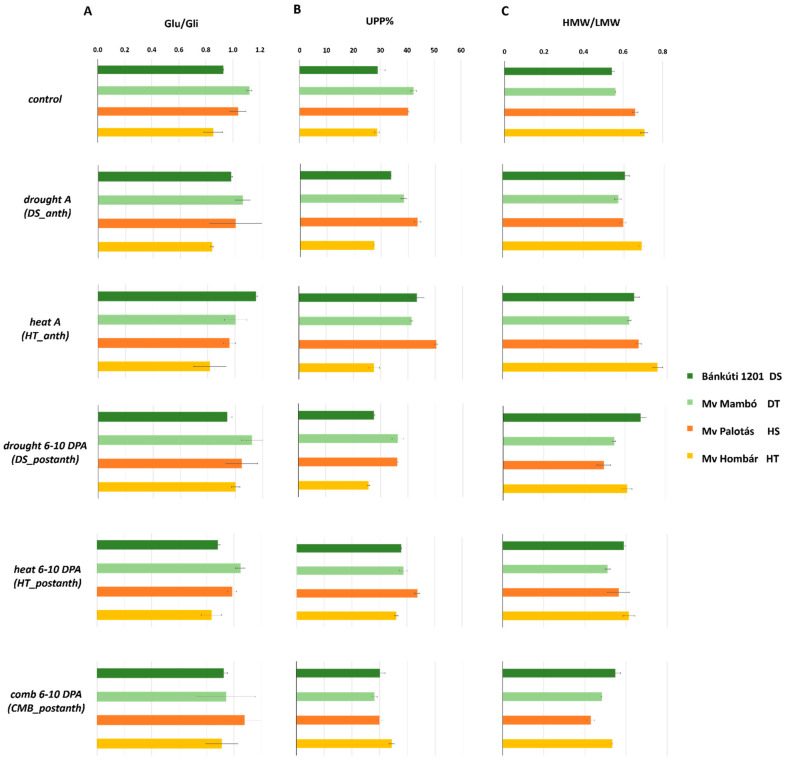
The alteration of Glu/Gli (**A**) and UPP% (**B**), as well as, HMW/LMW GS ratio (**C**) caused bydifferent abiotic treatments. UPP%—unextractable polymeric protein percentage; Glu/Gli—glutenin/gliadin ratio; HMW/LMW—high molecular weight glutenin/low molecular weight glutenin ratio. *DS_anth*: drought stress at anthesis; *HT_anth*: high temperature at anthesis; *CMB_anth*: combined stress at anthesis; *DS_postanth*: drought stress at post-anthesis; *HT_postanth*: high temperature at post-anthesis; *CMB_postanth*: combined stress at post-anthesis.

Protein content and composition data are shown in Table 2 and Figure 1, Figure 2 and Figure 3. The protein content of the examined wheat cultivars varied between 21.15 and 26.97%. Certain abiotic stress events at different growing stages generate changes in the overall protein content, as well as in protein composition.

Alteration of protein content and protein composition of the samples were in agreement with Juhász et al. [16] who found that the immunoreactive omega gliadin amount in the Hungarian bread wheat cultivars represented the lowest values among the gliadin subfractions of total protein under control conditions. In our study, the omega gliadin value fell between 1.07 and 1.42 g/100 g of wholemeal flour. The immunotoxic 33-mer-containing alpha gliadins represented the major fraction in all the cultivars, which means that under controlled temperature and weather conditions it falls between 4.22 and 5.19 g/100 g of wholemeal flour. Gamma gliadin fractions of the examined cultivars were between 3.29 and 6.72 g/100 g of wholemeal flour under controlled conditions. Under controlled conditions, the ratio of HMW to LMW subunits was found between 0.54 and 0.71. The Bx and Dx subunits showed the highest values among the HMW glutenin subunits, which varied between 0.88–1.86 g (g subunit/100 g of wholemeal flour) and 0.84–1.6 g (g subunit/100 g of wholemeal flour), respectively. The value of the Ax, By and Dy subunits fell between 0.39–0.53 g, 0.34–0.5 g and 0.39–0.54 g, respectively.

#### 2.2.1. Drought Stress

As early as 1971, Day and Barmore [19] reported that drought stress during the grain development period negatively affected the baking quality of spring wheat flour.

Based on the Jiang and Yu study [20], during grain filling, drought stress affects grain carbohydrates, storage protein synthesis and accumulation, consequently negatively affecting yield and quality. Besides, water shortage leads to a shorter grain filling period with decreased weight and number of seeds, associated with a significantly smaller size of starch granules due to less effective starch biosynthesis [21]. According to different studies [22,23], drought stress significantly increased the relative total seed storage protein content and changed the glutenin to gliadin ratio. The increase in protein and glutenin macro-polymer content results in improved dough properties and bread-making quality. In their study, significant upregulation of gliadins, HMW-GS and LMW-GS were detected under water deficit, among which alpha gliadins represented the greatest amount. Different research groups found that grain storage globulins, alpha gliadins, HMW and LMW glutenins, avenin-like proteins, and both allergy-related and celiac disease-associated omega and gamma gliadins, as well as 0.19 ATIs, serpins and 19 kDa globulins, were upregulated in conditions of water deficiency [24,25].

In the study of Phakela and co-workers [26], the effects of environmental stress conditions (drought and heat stress) on the alteration of gluten protein composition was investigated in six durum wheat cultivars. The severe drought stress treatment resulted in negative alteration of the HMW glutenins. They found that the LMW glutenins and gamma gliadin were decreased by the applied stress conditions. The applied heat and drought stress caused a significant increase in the alpha gliadin amount, which is in parallel with our results.

Our result showed that the ***DS_anth*** treatment led to the reduction in protein content by 6.31–20.25% compared to the control, except for the drought-tolerant cultivar. The glutenin/gliadin ratio showed a 2.7–5.95% decrease as well, while the drought-sensitive cultivar showed 4.3% elevated protein content. UPP% change was positive in Bánkúti 1201 and Mv Palotás by 16.58% and 7.9%, respectively, compared to the control; meanwhile, Mv Mambó and Mv Hombár showed a 9.43% and 4.9% decrease compared to the control. Water shortage at this early stage led to quality deterioration resulting from the alteration of the glutenin to gliadin amount (Table 2).

Compared to the control, ***DS_postanth*** resulted in a decrease of protein content in the heat-sensitive and heat-tolerant cultivars (14.97% and 4.94%, respectively). The drought-sensitive and drought-tolerant cultivars showed increased protein content (3.46% and 35.19%, respectively compared to the control). Positive changes in the Glu/Gli ratio were observed showing 0.02–1.13% values, and more increased value was detected in the heat tolerant cultivar, which accounts for the improvement in the quality. However, the UPP% was reduced by 4.37, 10.53, 11.02 and 14.35% in the drought sensitive, the heat sensitive, the heat tolerant and the drought tolerant cultivars, respectively.

According to the SE-HPLC results, ***DS_anth*** caused a 6.75 and 21.28% reduction in gliadin content at anthesis in drought-sensitive and heat-sensitive cultivars. Compared to the control, a 5.57 and 10.19% elevation of gliadin content was observed in the drought-tolerant and heat-tolerant cultivars, respectively. ***DS_postanth*** led to a 7.49–31.71% improvement of gliadin value, except for the heat-sensitive Mv Palotás. RP-HPLC results showed that ***DS_anth*** generated an 18.03% and 24.21% increase in the omega gliadin value in the case of the drought-sensitive (Bánkúti 1201) and heat-tolerant (Mv Hombár) cultivars compared to the control, but resulted in a 9.58% and 11.09% decrease in the drought tolerant (Mv Mambó) and heat-sensitive cultivar (Mv Palotás). ***DS_postanth*** resulted in a positive alteration, 28.79–52.62% in the omega gliadin content compared to the control, except for the heat-sensitive cultivar (Figure 1).

***DS_anth*** led to 1.36, 10.57, 19.77 and 81.02% increase in the alpha-gliadin fraction in the drought-sensitive, the drought-tolerant, the heat-sensitive and the heat-tolerant cultivars, respectively, compared to the control. In the case of ***DS_postanth***, 5.55–34.71% increase of alpha-gliadin content was observed as well, compared to the control.

***DS_anth*** caused a 6.17% increase in gamma gliadin in the heat-tolerant cultivar, but resulted in a 2.33–52.37% decrease in the other cultivars. Meanwhile, the ***DS_postanth*** led to a 41.41% decrease in the heat-sensitive cultivar, and a 2.38–26.75% increase in all other cultivars.

***DS_anth*** resulted in a 0.26–23.96% decrease in glutenin, while an 8.69–31.32% increase was observed in case of ***DS_postanth***. One exception was the heat-sensitive cultivar (Mv Palotás), which showed a 13.08% decrease in glutenin.

***DS_anth*** decreased the HMW to LMW glutenin ratio of the heat-sensitive and heat-tolerant cultivars by 9.8% and 2.67%, respectively, while drought-sensitive and drought-tolerant cultivars showed an increase of 11.26% and 1.8% respectively. ***DS_postanth*** resulted in a 2.46–24.72% reduction, except in the drought-sensitive cultivar, in which a 24.83% improvement was observed.

***DS_anth*** resulted in a decrease in the amount of the HMW glutenin Bx subunit in the heat-sensitive (by 38.29%) and heat-tolerant (by 10.54%) cultivars. Meanwhile, drought-sensitive and drought-tolerant cultivars showed an increase of 7.86% and 12.23%, respectively, compared to the control. ***DS_postanth*** caused a 10.30–37.87% improvement in the level of the Bx subunit compared to the control, except for one variety (Mv Palotás). ***DS_anth*** caused a reduction in the Dy subunit level in Mv Mambó (by 3.42%) and Mv Palotás (by 42.9%), while 6.77% and 1% increase was observed in Bánkúti 1201 and Mv Hombár, respectively. ***DS_postanth*** increased the level of the Dy subunit in the drought-tolerant and heat-tolerant cultivars by 25.58 and 35.18%, respectively, while a decrease of 0.02 and 36.39% of the Dy subunit was observed in the drought-sensitive and heat-sensitive cultivar. ***DS_anth*** resulted in a 16.4% and 20.62% elevation in the amount of the By subunit in drought-sensitive and heat-tolerant cultivars, respectively, while in drought-tolerant Mv Mambó and heat-sensitive Mv Palotás, a significant reduction was observed (41.14% and 46.27%,) compared to the control. Similar results were observed in case of ***DS_postanth***, so the amount of By subunit increased in drought-sensitive and heat-tolerant cultivars (22.73% and 30.99%) and, decreased in drought-tolerant and heat-sensitive cultivars (22.06% and 38.62%). The Dx subunit content in three of the investigated cultivars decreased by 2.38–22.01%, while it increased in the case of Bánkúti 1201. ***DS_postanth*** caused a reduction in Dx subunit amount in drought-sensitive (by 0.1%) and heat-sensitive cultivars (by 19.36%), while showing an increase in drought-tolerant (by 28.23%) and heat-tolerant cultivars (by 13.04%). ***DS_anth*** led to increased Ax subunit value in drought-tolerant and heat-sensitive cultivars (by 3.58% and 1.42%), while a decrease was observed in the drought-sensitive Bánkúti 1201 and heat-tolerant Mv Hombár (4.63% and 23.88%). ***DS_postanth*** significantly elevated the Ax subunit amount of the drought-sensitive and drought-tolerant cultivars (37.26% and 57.23%), while heat-sensitive and heat-tolerant cultivars showed a reduction of 18.06% and 15.18%, respectively (Figure 2).

#### 2.2.2. Heat Stress

Besides drought stress, higher temperature is another important abiotic factor that plays a crucial part in affecting crop development, grain filling and proteomic characteristics.

Iqbal and co-workers [27] summarized in a review that heat stress can modify the morphology, reduce grain size, plant height, grain growth duration, kernel number and kernel weight. In parallel, Hussain and co-workers [28] reported that high temperature stress led to decreased grain size and seed filling duration.

Other authors also reported a significant rise in grain protein content in response to heat stress [29,30,31,32,33,34,35].

Wang and co-workers [36] had summarized in a review the most important studies and results concerning the effects of different abiotic stress of wheat. It was concluded that abiotic stress generally induces complex proteomic changes in wheat grain, including decreased expression of proteins and pathways involved in normal growth and physiological processes, but up-regulated expression and function of those processes required for stress adaptation and tolerance, accompanied by significant reductions in kernel weight.

The studies discussed in the above-mentioned review revealed that heat and drought stress during flowering or post-anthesis increase the amounts of accumulating α- and ω-gliadins, as well as HMW-GS, but depending on genotypes, types of stresses and growth stages, exhibit different effects on the accumulation of LMW-GS [18,21,37,38,39]. The expression of α-gliadins was more strongly affected, indicating that the regulation of these proteins is more sensitive to abiotic stress. Other studies have confirmed that α-gliadins tend to be more sensitive to high temperature stress than other gliadin sub-fractions [6,37,40,41].

In heat stress, upregulation of serpins, 19 kDa globulin, alpha gliadins, and HMW glutenins can be observed, while 0.19 ATIs, ATI CM3, globulin-3, gamma gliadins, omega 1, 2, LMW glutenins, and avenin-like proteins are downregulated [18,24,25,39,42].

Wheat celiac-triggering protein expression levels depend on genotypes and cultivars and are primarily related to the extent of stress tolerance [43].

High temperature during the growing stages generally increased overall grain protein content, especially when accompanied with extended water shortage at anthesis and grain filling stages. Along with changes in the protein content, the glutenin to gliadin ratio and the proportion of the unextractable polymeric proteins (UPP%) also changed. This was expected to have a significant effect on the end-use quality, dough strength, flexibility, and elasticity.

From our study, it was revealed that the ***HT_anth*** treatment significantly increased the protein content by 5.88–12.58% compared to the control. Moreover, the glutenin to gliadin ratio showed a 4.29–10.48% decrease in relation to the control, while in the drought-sensitive cultivar, a 24.04% increase was observed. The UPP% was significantly raised in drought-sensitive and heat-sensitive cultivars (49.64% and 25.13%, respectively), while in drought-tolerant and heat-tolerant cultivars the UPP% decreased by 2.55% and 4%, compared to the control (Figure 3). It was concluded that early heat stress can lead to the improvement of both protein content and quality parameters, therefore the examined cultivars can successfully resist elevated temperature during at anthesis.

***HT_postanth*** resulted also in significant elevation in protein content (24.41–28.80%) compared to the control. The protein content was only reduced in the heat-sensitive cultivar in the case of ***HT_postanth***. Significant changes also occurred in the ratio of storage protein subfractions. The Glu/Gli ratio was altered negatively by 2.11–6.76% compared to the control; however, the UPP% showed an 8.54–31.38% increase (except for the drought-tolerant cultivar).

Concerning the gliadin composition, ***HT_anth*** led to a 12.62% and 47.18% improvement of the omega gliadin in drought-tolerant and heat-tolerant cultivars, respectively, while drought-sensitive and heat-sensitive cultivars showed a 19.98% and 9.8% decrease, compared to the control. ***HT_postanth*** caused a 20.37–28.37% increase in the amount of omega gliadins (except for the heat-sensitive cultivar).

The gamma gliadin content showed a 10.9–29.37% increase in three cultivars under ***HT_anth***, while the drought-sensitive Bánkúti 1201 showed a reduction in the gamma gliadin ratio by 6.84%. The ***HT_postanth*** increased the gamma gliadin content in all cultivars, except in the heat-sensitive cultivar.

The changes in the quantity of alpha-gliadins were the most diverse: an increase by 0.61–38.65% in three cultivars under ***HT_anth***, but an 8.23% decrease in the heat-sensitive cultivar. Similar results were obtained in case of ***HT_postanth*** a significant increase (28.49–84.66%) in three cultivars, and a 5.97% reduction in heat-sensitive Mv Palotás.

***HT_anth*** improved the ratio of the HMW to LMW by 6.58–17.46% in three examined cultivars, while the heat-sensitive Mv Palotás showed no change. Meanwhile, in ***HT_postanth***, a 9.08–14.68% reduction was observed, except in drought-sensitive Bánkúti 1201.

Regarding the HMW glutenin composition, ***HT_anth*** resulted in a 6–13.86% increase in the Bx subunit value in three of the four examined cultivars (except in heat-sensitive Mv Palotás). Similar results were detected in the case of ***HT_postanth***, with the amount of the Bx subunit showing 4.96–25.99% improvement, but decreased values were observed in the heat-sensitive Mv Palotás. The ***HT_anth*** caused 6.73% and 8.61% reduction in the Dy subunit content in the drought-tolerant and heat-sensitive cultivars, while the drought-sensitive Bánkúti 1201 and the heat-tolerant Mv Hombár showed an increase of 22.14% and 72.97% compared to the control. Similar results were observed in the Dy subunit value in ***HT_postanth***. The ***HT_anth*** improved the amount of the By subunit by 0.25–43.64%. The ***HT_postanth*** showed an 8.9% and 52.05% decrease in the drought-tolerant and heat-sensitive cultivars, while a 26.41 and 33.61% improvement was observed in the heat-tolerant and drought-sensitive cultivars. A decrease was observed in the level of the Dx subunit in the drought-tolerant and heat-sensitive cultivars (11.46% and 8.82%, respectively) under ***HT_anth***, while in drought-sensitive Bánkúti 1201 and heat-tolerant Mv Hombár, a 26.92% and 20.13% increase was observed, compared to the control. The ***HT_postanth*** caused an increase of 18.02–43.61% in the Dx subunit in three of the four investigated cultivars, except in heat-sensitive Mv Palotás. ***HT_anth*** reduced the Ax subunit amount of the drought-tolerant and heat-tolerant cultivars by 17.06% and 2.28%, while an increase was observed in the drought-sensitive Bánkúti 1201 and heat-sensitive Mv Palotás (14.15% and 4.48%, respectively). An increase of 13.96–28.05% was observed in ***HT_postanth***, compared to the control, except in the heat-sensitive Mv Palotás (Figure 3). It clearly seems that the heat-sensitive cultivar Mv Palotás has very different abiotic stress-resistant capacity/properties compared to the other three examined cultivars, and this is reflected in the yield, gliadin and the HMW subunit content also.

#### 2.2.3. Combined Stress

Balla et al. [44] studied the effect of high temperature and drought on the composition of gluten proteins in winter wheat varieties. They found that drought has a much greater influence on yield and quality than high temperature.

In this study, it was observed that the ***CMB_postanth*** treatment caused a 5.13–48.05% increase in protein content. The Glu/Gli ratio decreased in drought-sensitive and drought-tolerant cultivars by 0.53 and 16.07%, while increased in heat-sensitive and heat-tolerant cultivars by 3.78 and 6.66%, respectively. The UPP% decreased in drought-tolerant and heat-sensitive cultivars by 33.64% and 25.75%, while a 4.06% and 19.6% improvement was observed in drought-sensitive and heat-tolerant cultivars, compared to the control. Although improvement was detected in protein content, ***CMB_postanth*** caused a significant reduction in quality.

The increase in the gliadin subfractions was proven in all the four Hungarian cultivars under **CMB_postanth***. **CMB_postanth*** significantly improved the omega gliadin amount by 12.77–36.78% compared to the control, except in the heat-sensitive cultivar. The omega gliadin content of wheat is highly affected by the elevated temperature, and the combination with water shortage is able to increase its ratio in relation to gamma- and alpha-gliadin subfractions.

The ***CMB_postanth*** increased the gamma gliadin amount by 3.79–64.57% compared to the control.

In summary, the results show that not only the omega and gamma gliadin levels were increased under ***CMB_postanth***, but the alpha-gliadin content also increased by 11.62–69.49% depending on the variety. The least effect was detected in the drought-sensitive Bánkúti 1201, while the most severe impact was observed in the drought-tolerant cultivar.

The HMW to LMW glutenin ratio decreased by 14.26–35.33% compared to the control under ***CMB_postanth***, except in the drought-sensitive cultivar where a 0.65% improvement was detected. Among HMW glutenin subunits, the Bx level was reduced by 13.69% and 28.66% in the case of the drought-tolerant and heat-sensitive cultivars, respectively, under ***CMB_postanth***, while the drought-sensitive and heat-tolerant cultivars showed an 18.57% and 54.57% elevation, compared to the control. The Dy subunit level decreased in Mv Mambó (by 18.96%) and Mv Palotás (by 23.05%), but in Bánkúti 1201 and heat-tolerant Mv Hombár, enhanced values were observed (0.39% and 75.10%, respectively). ***CMB_postanth*** led to a reduction of the By subunit amount in the drought-tolerant and heat-sensitive cultivars (25.16% and 35.18%, respectively), while an improvement of 24.93% and 40.96% was observed in drought-sensitive and heat-tolerant cultivars. Drought-sensitive and heat-tolerant cultivars showed an increased level of Dx subunit (18.99% and 38.86%, respectively), but 8.89% and 11.95% reduction was detected in drought-tolerant and heat-sensitive cultivars. The Ax subunit amount increased by 5.33–19.29% compared to the control under ***CMB_postanth***, except in heat-sensitive Mv Palotás.

Changes in protein parameters caused by the different abiotic treatments were evaluated by ANOVA (Table 2 and Table 3). In the light of observations taken for the physiological parameters (Table 1), it is not surprising that significant changes have been observed for each protein parameter caused by treatments among the four cultivars with different abiotic sensitivity. However, the sensitivity of different parameters can be differentiated based on the F values; gliadin content and protein content seem to be more sensitive to abiotic conditions than the HMW to LMW ratio, followed by the glutenin to gliadin ratio and glutenin content. In gliadin subclasses, the sensitivity sequence is ω > γ > α/β and Ax > Bx~Dx > Dy~By for HMW GS. The results of these experiments fully correspond to the data published in the scientific literature.

### 2.3. Effect of Abiotic Stress to Immunoreactive R5 and G12 Peptide Content

In general, high temperatures increase celiac disease-associated protein and gluten protein content, mainly due to the significant decrease in the amount of starch [38].

Dubois and co-workers [45] investigated the expression of the α-gliadin epitopes of north-western European spelt landraces using quantitative PCR, and significant variations were identified in the content of epitope transcripts between accessions. However, each individual accession showed good relative stability in the content of epitopes across four harvest years.

In our previous study [16], two Norwegian bread wheat cultivars, Bjarne and Berserk, were investigated in order to determine the influence of temperature on grain allergen and antigen-response proteins. However, the heat tolerance ability of the northern bread wheat varieties cannot be comparable with the Hungarian wheat cultivars adapted to the unique microclimate of the Carpathian Basin. According to climate prediction models, short-term temperature extremities and water shortages will become more frequent.

The portion of immunoreactive ω-gliadins in Berserk, Bjarne, and Chinese Spring wheat cultivars comprised 3.7, 6.6, and 5.4%, respectively, of total protein under normal Norwegian conditions (20 °C/16 °C day/night). The effect of high temperature was more significant in Chinese Spring and Berserk, with an increase of 25.6 and 13.3%, respectively, while a small rise of 3.7% was observed in Bjarne. These data showed that strong ω-gliadin antigens were expressed in high quantities in grains and that their expression level was greatly affected by temperature.

Under normal temperature conditions, the immunotoxic 33-mer-containing α-gliadins comprised 2.7–3.1% of the total protein content in all three cultivars. The amount of the α-gliadin fraction increased with 25 to 33% under high temperatures. Bjarne, a high-protein Norwegian cultivar showed significantly different expression patterns in these major allergens. However, the effect of high temperature was not significant. Under normal conditions, Bjarne produced a stronger G12 mAb response, while Berserk showed a lower G12 mAb response. High temperature led to a moderate decrease in all three cultivars. The R5 mAb primarily detects QQPFP peptides that are present in 67% of α-gliadins, 90% of γ-gliadins, and 28% of ω-gliadin sequences in Chinese Spring. Bjarne and Berserk showed 30% and 16% less R5 peptide content compared to Chinese Spring under normal conditions. High-temperature conditions had a slightly negative impact on the R5 mAb response.

Our institute is currently applying the same techniques in monitoring the effects of growing conditions on protein composition, as well as the relationships between the protein composition and the techno-functional properties, of our high-yielding and quality modern wheat varieties. Based on climate model forecasts [46,47], in this ongoing study, the abiotic stress factors are investigated with elevated atmospheric carbon dioxide complementation, combined with different amounts of nitrogen fertilizer in a FACE (free carbon dioxide enrichment) ring.

In this study, the R5 and G12 reactive protein content of the investigated wheat varieties was monitored with commercially available sandwich ELISA tests. The ELISA assay is a widely used method that gives quantified information on immunoreactive epitope contamination in food products. It is therefore used to detect levels of gluten-like proteins in cereal grains.

In this study, R-Biopharm RIDASCREEN^®^ Gliadin R5 and ROMER AgraQuant^®^ Gluten G12^®^ sandwich ELISA tests were used for monitoring of the gluten-related harmful peptide contents of the samples.

The R5 mAb was developed against the peptide QQPFP, characteristic in rye ω-secalin, barley hordeins, and wheat gliadins. R5 also recognizes homologous peptides such as LQPFP, QLPYP, QLPTF, QQSFP, QQTFP, PQPFP and QQPYP, however, with weaker reactivity [48,49]. The monoclonal antibody G12 was raised against the QPQLPY peptide [50], present in the alpha 2-gliadin 33-mer immunotoxic epitope in wheat (LQLQPFPQPQLPYPQPQLPYPQPQLPYPQPQPF), which contains three different core-epitopes (PFPQPQLPY, PQPQLPYPQ, and PYPQPQLPY), of which two are present in duplicate.

**Figure 4:**
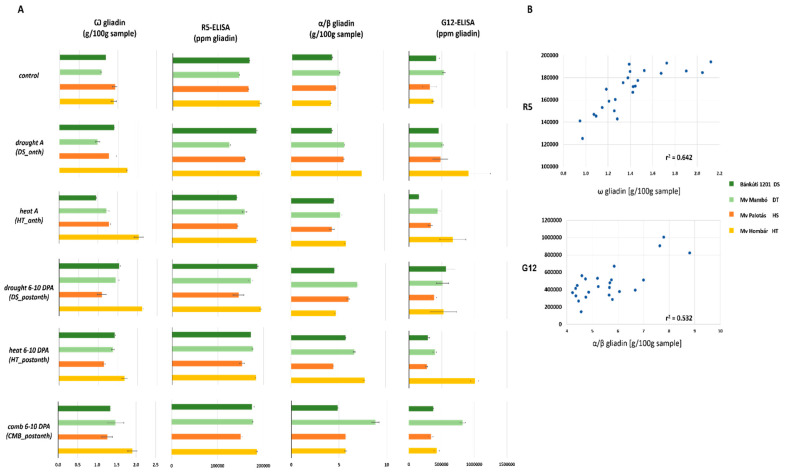
Comparison (**A**) and relationship (**B**) of gliadin levels determined by SE-HPLC as well as R5 and G12 ELISA tests. *DS_anth*: drought stress at anthesis; *HT_anth*: high temperature at anthesis; *CMB_anth*: combined stress at anthesis; *DS_postanth*: drought stress at post-anthesis; *HT_postanth*: high temperature at post-anthesis; *CMB_postanth*: combined stress at post-anthesis.

According to R5 ELISA test results in this study (Table 4 and Figure 4), the heat-tolerant cultivar (Mv Hombár) showed the highest content of gliadin, followed by the drought-sensitive (Bánkúti 1201), heat-sensitive (Mv Palotás), and drought-tolerant cultivars (Mv Mambó). Environmental stress treatments caused significant changes in the gliadin content of the cultivars compared to the control. Comparison of the F values originating from the ANOVA analysis showed that the combined heat and drought treatments had the most significant effect on the gliadin levels among the different abiotic treatments (Table 3). The F value for the cultivar x treatment interaction was ten times higher in the case of R5 results than for the G12. This was probably because of the larger inter-cultivar differences in ω gliadin alterations than in the α/β gliadin alterations. There was a good correlation between the gliadin subclass values measured by RP-HPLC and the corresponding ELISA data (Figure 4); the correlation coefficient between ω gliadin content and R5 data was 0.642, while between the α/β gliadin content and G12, it was 0.532.

***DS_anth*** caused a 14.71% and 3.85% decrease in the content of gluten compared to the control in the heat-sensitive and the drought-tolerant cultivars, while an increase was observed in the drought-sensitive (9.41%) and heat-tolerant cultivar (0.51%).

***HT_anth*** caused a decrease in the content of gluten compared to the control in all the cultivars (3.88–16.8%), with the exception of the drought-tolerant cultivar.

***DS_postanth*** caused an increase in gluten content compared to the control in all of the cultivars by 1.15–17.33%, with the exception of the heat-sensitive cultivar.

***HT_postanth*** generated a remarkable increase in gluten content compared to the control in the drought-sensitive and drought-tolerant cultivars (1.44% and 22.37%, respectively). Meanwhile, ***HT_postanth*** led to the reduction of gluten value compared to the control in the heat-sensitive and heat-tolerant cultivars by 8.17% and 4.3%, respectively.

***CMB_postanth*** increased gluten content by 3.47% and 20.18% in the drought-sensitive and drought-tolerant cultivars, respectively, and decreased gluten value by 9.93% and 3.14% in heat-sensitive and heat-tolerant cultivars, respectively.

The G12 ELISA test showed that the drought-tolerant (Mv Mambó) variety had the highest gluten content, followed by the drought-sensitive (Bánkúti 1201), heat-tolerant (Mv Hombár) and heat-sensitive (Mv Palotás) cultivars. The applied environmental stress treatments caused significant differences in the G12 reactive protein content in all of the investigated cultivars compared to the control.

***DS_anth*** caused an increase in the G12 reactive protein content in the drought-sensitive, heat-sensitive and heat-tolerant cultivars by 8.6, 51.92 and 146.74%, respectively, but in the drought-tolerant cultivar, a 3.39% reduction of gluten content was observed compared to the control.

***HT_anth*** caused an increase in gluten in heat sensitive and heat-tolerant cultivars by 4.06% and 82.71%, respectively, compared to the control. Meanwhile, a decrease in gluten content was observed in drought-sensitive and drought-tolerant cultivars by 64.78% and 17.75%, respectively, compared to the control.

Gluten values showed an increase of 20.33–43.04% in almost all cultivars, due to ***DS_postanth***, but in the drought-tolerant cultivar, a 3.48% reduction was observed.

***HT_postanth*** caused a reduction in gluten content in three of the four examined cultivars by 14.66–30.4%. However, a significant increase was observed in gluten content in the heat-tolerant cultivar (174.25%) compared to the control.

***CMB_postanth*** resulted in a 10.4% reduction in gluten content in the drought-sensitive cultivar. The heat-sensitive and heat-tolerant cultivars showed a 6.88% and 16.16% increase, while the drought-tolerant cultivar showed a significant increase of 55.36% in gluten content.

The variance in gluten levels measured with R5 and G12 could be explained by the different target sequences of the antibodies.

## 3. Materials and Methods

### 3.1. Plant Material

Hungarian bread wheat cultivars (*Triticum aestivum* ssp. *aestivum*), drought-sensitive Bánkúti 1201, drought-resistant Mv Mambó, heat-sensitive Mv Palotás, and heat-resistant Mv Hombár were provided by the Cereal Gene Bank Agricultural Research Centre, Martonvásár, Hungary. Mv Mambó, Mv Palotás and Mv Hombár varieties were bred in Martonvásár, Hungary.

### 3.2. Growing Conditions and Abiotic Stress Treatments

Seeds were germinated for three to five days on a wet filter under dark conditions at 24 °C, and were grown in a vernalisation chamber (Conviron J-01) in Jiffy pots at 4 °C for 6 weeks.

Thereafter, the plants were grown in plastic pots filled with sterilized chernozem soil, in a Conviron PGV-36 phytotron chamber, in a 16/8 h light-dark photoperiod (Controlled Environments, Winnipeg, MB, Canada), according to the Carpathian Basin climate characteristic, photoperiod season, and temperature [51]. Six treatments as well as controls were used in all varieties; drought, heat and combined stress at anthesis and in 6–10 DPA.

The detailed parameters used as heat stress conditions are shown in Supplementary Table S3. A one-week long period of total water withdrawal was applied at the drought stress treatments both at anthesis and in the 6–10 DPA phenological stage.

Soil moisture stress was expressed in percentage compared to the control. The soil moisture in the control was between the wilting point-field capacity (WP-FC) of about 35%, which reduced to about 15% at the end of the one-week long total water withdrawal. The tests in Martonvásár were carried out on good quality soil, therefore WP and FC could be estimated. Soil water content (SWC) in classic volumetric m^3^/m^3^.

### 3.3. Protein Content

The crude protein content was determined by the Dumas method in triplicate, using a nitrogen conversion factor of 5.7 and an adaptation of the AOAC Official Method [52] on an automated protein analyser (LECO FP-528, LECO Corporation, St. Joseph, MO, USA).

### 3.4. RIDASCREEN R5 and ROMER G12 Sandwich ELISA Tests

R-Biopharm RIDASCREEN^TM^ Gliadin R5 (catalogue number: R7001, R5 monoclonal antibody, sandwich format, LoD: 0.5 mg/kg gliadin or 1 mg/kg gluten, LoQ: 2.5 mg/kg gliadin or 5 mg/kg gluten, R-Biopharm AG, Darmstadt, Germany) sandwich enzyme immunoassay and the ROMER AgraQuant^TM^ Gluten G12^TM^ (catalogue number: COKAL0200, G12 monoclonal antibody, sandwich format, LoD: 2 mg/kg gluten, LoQ: 4 mg/kg gluten, Romer Labs Diagnostic GmbH, Tulln, Austria) sandwich enzyme assay were used to determine the harmful epitope content of the prolamin extracts.

Prolamin extraction and dilution of *Triticum aestivum* samples was performed in four replicates according to the manufacturer’s instructions. The bread wheat extracts were diluted to 1:2,500,000 (in R5), and to 1:5000 (in G12) final concentrations to ensure sufficient sensitivity even at lower protein levels. ELISA assays were performed as outlined in the manuals provided by the manufacturers; the cubic spline algorithm was used for the standard curve construction. Results were corrected by the dilution factor used for the flour samples.

### 3.5. Size Exclusion High Performance Liquid Chromatography (SE-HPLC)

SE-HPLC was used to determine the glutenin, gliadin and albumin + globulin contents, using a modification of the Batey et al. [53] method, as described by Rakszegi et al. [54].

Ten milligrams of flour were suspended in 1 mL 0.5% (*w*/*v*) sodium dodecyl sulphate (SDS) in phosphate buffer (pH 6.9) and sonicated for 15 s. After centrifugation, the supernatant was filtered through a 0.45 μm PVDF filter. Analyses were performed through a Phenomenex BIOSEP-SEC 4000 column (300 × 7.8 mm, 5 µm, 500 Å) in acetonitrile buffer 0.05% (*v*/*v*) trifluoroacetic acid and 0.05% (*v*/*v*) acetonitrile] with a running time of 10 min (2 mL/min flow rate). Proteins were detected by absorption at 214 nm.

Protein class contents were expressed as [% of the sample] by combining protein content and data from the SE-HPLC separation. The effect of different abiotic treatments on protein content in the three protein classes has been determined by comparing the data as a percentage of the corresponding values of the control.

Unextractable polymeric protein percentage (UPP%) was determined using the method of Gupta et al. [55], applying a two-step extraction procedure.

### 3.6. Reversed-Phase High Performance Liquid Chromatography (RP-HPLC)

Proteins were extracted from flour (60 mg) using 70% ethanol and vortexed for 30 min in a horizontal vortex, (Inc. Vortex-Genie^®^ 2, MO BIO Laboratories, New York, NY, USA). Samples were centrifuged for 15 min at 13,000 rpm using an Eppendorf Centrifuge 5424. The supernatant was aspirated through a 0.45 µL filter and dispensed into an HPLC glass vial. The protein extracts were separated using an Agilent 1200 LC system (Agilent Technologies, Santa Clara, CA, USA) using the Larroque et al. method [56]. In brief, 10 µL of extract were injected into a C18 reversed-phase Zorbax 300SB-C18 column (4.6 × 150 mm, 5 µm, 300 Å, Agilent Technologies) maintained at 60 °C. The eluents used were ultrapure water (solvent A) and acetonitrile (solvent B), each containing 0.1% TFA (Trifluoroacetic acid, HPLC grade, Sigma Aldrich, St. Louis, MO, USA). The flow rate was adjusted to 1 mL/min. Protein was separated using a linear gradient from 21% to 47% of solvent B in 55 min and detected by UV absorbance at 210 nm. Each sample was sequentially injected three times for technical replication. RP-HPLC peak areas (expressed in arbitrary units, AU) under the chromatograms were used to calculate gliadin amounts. Retention times for ω-gliadins were between 15–30, for α-gliadins 30–40, and for γ-gliadins 40–55 min [57].

Gliadin content [% of the sample] was determined from data derived from the SE-HPLC separation of the total protein content multiplied by the protein content of the sample. α/β, γ and ω gliadin contents [% of the sample] were calculated from the data derived from the RP-HPLC separation of gliadin proteins, multiplied by the gliadin content of the total protein, determined by the SE-HPLC separation and multiplied by the protein content of the samples.

A modification of the RP-HPLC method of Marchylo et al. [58] was used to determine the relative amounts of the HMW glutenin subunits. The gliadins were extracted using 70% (*v*/*v*) ethanol and vortexed for 30 min in a horizontal vortex. Samples were then centrifuged for 4 min at 13,200 rpm. The supernatant was aspirated using a 0.45 µm PVDF filter and dispensed into an HPLC glass vial. The residuals were washed twice with 50% (*v*/*v*) propan-1-ol, vortexed for 30 min, and centrifuged for 4 min at 13,200 rpm. The glutenin polymers were then reduced with buffer (50% (*v*/*v*) propan-1-ol, 2 M urea and 0.2 M Tris-HCl, pH 6.6) containing 1% (*w*/*v*) reducing agent, dithiothreitol (DTT) for 1 h at 60 °C, with mixing in every 10 min. Samples were alkylated *w/v* with 4-vinylpyridine for a further 15 min at 60 °C, with mixing every 10 min. The mixtures were centrifuged for 4 min at 13,200 rpm. The supernatant was aspirated using a 0.45 µm PVDF filter and dispensed into an HPLC glass vial. The protein extracts were separated on a Supercosil LC-308 column (300 A, 3.5% carbon, 5 mm, 5 × 4.6).

The HMW GS protein content was expressed as [% of the sample] by combining protein content, glutenin content derived from SE-HPLC, and the data from the RP-HPLC separation. The effects of different abiotic treatments on the amounts of the five glutenin subunits were determined by comparing the data as a percentage of the corresponding values of the control.

### 3.7. Statistical Analyses

Basic statistics and ANOVA tests, as well as multiple comparisons of mean values based on the least significant difference (LSD) by Student’s *t*-tests, were carried out as implemented in the NCSS 2021 Statistical Software (2021) NCSS 2021 Statistical Software (2021) (NCSS, LLC, Kaysville, UT, USA); significance levels were set to *p* < 0.05.

## 4. Conclusions

The combined use of cereal chemistry and immune analytics provides a better understanding of the effect of abiotic stressors on flour quality and the quantitative change in celiac-related proteins. The genetic variability and the determination of the quality parameters can serve as a good base against the challenges of climate change. The quantitation of the protein classes has a role in bread-making quality, and understanding their environmental stability will enhance food security in the future.

Due to climate change, it is becoming more frequent that drought stress paired with a high temperature occurs in the Carpathian Basin, so it is not enough to focus on preparing and breeding only against individual stresses. Climate chamber experiments were very useful in investigating specific heat, drought and combined abiotic stress effects. Determining their influence on yield and protein composition provides valuable information for establishing altered breeding strategies suitable for developing superior bread wheat cultivars in the Carpathian Basin in a changing climatic environment. In this study, the yield was increased by the individual high temperature stress, while significantly reduced by the combined drought and heat stress treatment. In agreement with the above-mentioned studies, the alpha gliadins were increased by all stress treatments. The timing of the applied stress treatment is an important aspect. The HMW glutenin, omega- and gamma gliadins caused by the different abiotic stress treatments showed different alterations depending on the timing of the applied treatments. It is possible that the currently bred wheat varieties will not be competitive in the future and unable to adapt to changing environmental conditions, so the breeding of modern, stress adaptive, early ripening cultivars is important for climate-smart agriculture. As a direct continuation of this study, intensive research is in progress to improve our knowledge about climate-related abiotic stresses, aiming to limit the yield and quality drops of wheat production in the Carpathian Basin caused by individual or combined heat, drought stresses. Our investigations are completed with the monitoring of nitrogen fertilization and elevated atmospheric carbon dioxide-related effects combined with abiotic stresses.

## Figures and Tables

**Table 1:** The alteration of physiological parameters caused by the different abiotic conditions.

	Plant Height (cm)		Number of Spikelets Per Main Spike		Number of Grains Per Main Spike		Weight of Grains Per Main Spike (g)		Summarized Weight of Grains (g)		Thousand Kernel Weight	
**Mean**	38.21		17.79		33.30		0.69		1.61		20.37	
**F for VARIETIES**	8081.42	***	11.97	***	3051.50	***	929.52	***	767.17	***	848.35	***
**F for TREATMENTS**	366.53	***	1.10		18.29	***	9.29	***	5.33	***	7.31	***
F for VAR*TRE	7.60	***	6.14	***	3.59	***	17.83	***	33.64	***	25.74	***
**LSD (cal by t)**	3.14		0.81		2.95		0.17		0.25		3.27	
**Bánkúti 1201 (DS)**	63.9	**C**	16.8	**A**	28.4	**A**	0.87	**C**	2.11	**C**	30.61	**C**
**Mv Mambó (DT)**	29.3	**A**	19.1	**B**	33.0	**B**	0.62	**A**	1.31	**A**	17.87	**AB**
**Mv Palotás (HS)**	30.5	**A**	17.1	**A**	31.9	**B**	0.51	**A**	1.40	**A**	15.45	**A**
**Mv Hombár (HT)**	33.1	**B**	18.1	**B**	39.3	**C**	0.81	**B**	1.74	**B**	19.40	**B**
**LSD (cal by t)**	3.27		0.74		3.14		0.17		3.74		6.14	
**Control**	41.8	**D**	18.2	**A**	35.5	**D**	0.80	**D**	1.96	**C**	23.21	**C**
**Drought A (*DS_anth*)**	37.2	**B**	17.6	**A**	31.4	**B**	0.61	**C**	1.10	**B**	18.23	**B**
**Heat A** **(*HT_anth*)**	34.8	**B**	18.5	**A**	37.8	**D**	1.11	**E**	3.01	**D**	29.35	**D**
**Comb A (*CMB_anth*)**	30.5	**A**	17.6	**A**	26.5	**A**	0.16	**A**	0.23	**A**	6.04	**A**
**Drought 6–10 DPA** **(*DS_postanth*)**	40.0	**C**	18.0	**A**	33.9	**C**	0.69	**C**	1.45	**B**	21.00	**C**
**Heat 6–10 DPA (*HT_postanth*)**	39.2	**C**	17.3	**A**	36.4	**D**	0.97	**D**	2.63	**D**	27.98	**D**
**Comb 6–10 DPA (*CMB_postanth*)**	42.5	**D**	17.5	**A**	30.5	**B**	0.39	**B**	0.61	**B**	13.88	**B**

*** indicate significant relationships (*p* < 0.001). Capital letters indicate significantly different mean values, comparing them to LSD (least significant difference by Student’s *t*-test). A: anthesis; 6–10 DPA: 6–10 day post-anthesis. DS—drought sensitive; DT—drought tolerant; HS—heat sensitive; HT—heat tolerant. *DS_anth*: drought stress at anthesis; *HT_anth*: high temperature at anthesis; *CMB_anth*: combined stress at anthesis; *DS_postanth*: drought stress at post-anthesis; *HT_postanth*: high temperature at post-anthesis; *CMB_postanth*: combined stress at post-anthesis.

**Table 2:** The alteration of protein composition caused by the different abiotic conditions.

	Protein%		Glu%		Gli%		GLU/GLI		UPP		HMW/LMW	
**Mean**	25.77		10.55		11.77		0.98		35.41		0.59	
**F for VARIETIES**	1254.30	***	133.54	***	2098.38	***	611.10	***	236.10	***	72.00	***
**F for TREATMENTS**	4677.13	***	60.92	***	1152.01	***	64.71	***	116.30	***	80.59	***
**F for VAR × TRE**	946.18	***	54.41	***	312.58	***	100.01	***	39.78	***	13.90	***
**LSD (cal by t)**	0.13		0.12		0.11		0.01		0.86		0.015	
**Bánkúti 1201 (DS)**	23.89	**A**	10.05	**A**	9.35	**A**	0.97	**B**	33.53	**B**	0.60	**C**
**Mv Mambó (DT)**	26.93	**C**	11.10	**D**	12.14	**B**	1.05	**D**	37.51	**C**	0.55	**A**
**Mv Palotás (HS)**	25.05	**B**	10.76	**C**	12.19	**B**	1.02	**C**	40.65	**D**	0.57	**B**
**Mv Hombár (HT)**	27.22	**D**	10.29	**B**	13.41	**C**	0.87	**A**	29.94	**A**	0.65	**D**
**LSD (cal by t)**	0.17		0.14		0.13		0.01		1.06		0.018	
**Control**	22.90	**B**	10.43	**B**	10.47	**B**	0.98	**C**	35.10	**B**	0.62	**C**
**Drought A (*DS_anth*)**	20.83	**A**	10.47	**B**	10.03	**A**	0.96	**B**	35.68	**B**	0.62	**C**
**Heat A (*HT_anth*)**	25.19	**D**	10.21	**A**	11.76	**D**	0.98	**C**	40.62	**D**	0.67	**D**
**Comb A (*CMB_anth*)**	32.98	**G**										
**Drought 6–10 DPA (*DS_postanth*)**	23.65	**C**	11.20	**D**	11.37	**C**	1.03	**D**	31.36	**A**	0.58	**B**
**Heat 6–10 DPA (*HT_postanth*)**	26.28	**E**	10.20	**A**	12.69	**E**	0.94	**A**	39.09	**C**	0.57	**B**
**Comb 6–10 DPA (*CMB_postanth*)**	28.59	**F**	10.81	**C**	14.31	**F**	0.96	**B**	30.60	**A**	0.50	**A**

*** indicate significant relationships (*p* < 0.001). Capital letters indicate significantly different mean values. Capital letters indicate significantly different mean values, comparing them to LSD (least significant difference by Student’s *t*-test). DS—drought sensitive; DT—drought tolerant; HS—heat sensitive; HT—heat tolerant. *DS_anth*: drought stress at anthesis; *HT_anth*: high temperature at anthesis; *CMB_anth*: combined stress at anthesis; *DS_postanth*: drought stress at post-anthesis; *HT_postanth*: high temperature at post-anthesis; *CMB_postanth*: combined stress at post-anthesis.

**Table 3:** The alteration of protein composition caused by the different abiotic conditions based on the RP-HPLC analyses.

	Omega		Alpha/Beta		Gamma		Bx		Dy		By		Dx		Ax	
	Gliadin%						HMW-GS%									
**Mean**	1.892		5.883		3.996		1.22		0.46		0.41		1.25		83.62	
**F for VARIETIES**	2834.29	***	949.75	***	1265.57	***	92.62	***	47.74	***	103.44	***	104.60	***	9.80	***
**F for** **TREATMENTS**	456.71	***	284.66	***	816.08	***	11.10	***	15.96	***	18.44	***	11.38	***	17.88	***
F for VAR*TRE	108.55	***	105.20	***	252.15	***	31.14	***	20.30	***	11.84	***	12.62	***	11.8	***
**LSD (cal by t)**	0.06		0.06		0.04		0.05		0.02		0.03		0.06		0.03	
**Bánkúti 1201 (DS)**	1.17	**A**	4.75	**A**	3.43	**A**	1.03	**A**	0.45	**B**	0.45	**C**	0.94	**A**	0.43	**B**
**Mv Mambó (DT)**	1.46	**B**	6.36	**C**	4.32	**B**	1.14	**B**	0.46	**B**	0.29	**A**	1.34	**CB**	0.59	**D**
**Mv Palotás (HS)**	1.41	**B**	6.95	**C**	3.83	**A**	1.41	**D**	0.41	**A**	0.38	**B**	1.39	**C**	0.40	**A**
**Mv Hombár (HT)**	3.53	**C**	5.48	**B**	4.41	**B**	1.30	**C**	0.54	**C**	0.52	**D**	1.30	**B**	0.53	**C**
**LSD (cal by t)**	0.07		0.07		0.04		0.06		0.03		0.03		0.07		0.03	
**Control**	1.47	**A**	5.31	**A**	3.70	**A**	1.24	**B**	0.45	**B**	0.41	**B**	1.21	**B**	0.47	**AB**
**Drought A (*DS_anth*)**	1.54	**B**	5.11	**A**	3.38	**A**	1.08	**A**	0.40	**A**	0.35	**A**	1.11	**A**	0.44	**A**
**Heat A (*HT_anth*)**	1.75	**C**	6.12	**B**	3.89	**A**	1.28	**B**	0.53	**C**	0.51	**C**	1.26	**CB**	0.47	**AB**
**Comb A (*CMB_anth*)**																
**Drought 6–10 DPA (*DS_postanth*)**	1.75	**C**	5.59	**AB**	4.03	**B**	1.22	**B**	0.47	**B**	0.39	**B**	1.26	**CB**	0.55	**D**
**Heat 6–10 DPA (*HT_postanth*)**	1.91	**D**	6.42	**B**	4.36	**C**	1.24	**B**	0.45	**B**	0.39	**B**	1.36	**D**	0.52	**C**
**Comb 6–10 DPA (*CMB_postanth*)**	2.95	**E**	6.74	**C**	4.63	**D**	1.26	**B**	0.48	**B**	0.41	**B**	1.28	**C**	0.49	**BC**

*** indicate significant relationships (*p* < 0.001). Capital letters indicate significantly different mean values, comparing them to LSD (least significant difference by Student’s *t*-test). DS—drought sensitive; DT—drought tolerant; HS—heat sensitive; HT—heat tolerant. *DS_anth*: drought stress at anthesis; *HT_anth*: high temperature at anthesis; *CMB_anth*: combined stress at anthesis; *DS_postanth*: drought stress at post-anthesis; *HT_postanth*: high temperature at post-anthesis; *CMB_postanth*: combined stress at post-anthesis.

**Table 4:** The alteration of the immune reactive R5 and G12 peptides caused by the different abiotic conditions.

	R5		G12	
	ppm Gliadin% of the Control		ppm Gliadin% of the Control	
**Mean**	100.57		127.17	
**F for VARIETIES**	173.39	***	29.79	***
**F for TREATMENTS**	26.90	***	4.32	***
F for VAR*TRE	31.69	***	3.84	***
**LSD (cal by t)**	2.70		2.97	
**Bánkúti 1201 (DS)**	101.50	**C**	101.77	**B**
**Mv Mambó (DT)**	97.27	**B**	111.62	**C**
**Mv Palotás (HS)**	110.86	**D**	90.97	**A**
**Mv Hombár (HT)**	92.79	**A**	200.69	**D**
**LSD (cal by t)**	2.11		1.41	
**Control**	100.00	**B**	100.00	**B**
**Drought A (*DS_anth*)**	98.35	**B**	144.24	**E**
**Heat A (*HT_anth*)**	92.92	**A**	97.16	**A**
**Comb A (*CMB_anth*)**	107.54	**D**	191.60	**E**
**Drought 6–10 DPA (*DS_postanth*)**	103.43	**D**	121.61	**D**
**Heat 6–10 DPA (*HT_postanth*)**	101.99	**C**	120.35	**C**
**Comb 6–10 DPA (*CMB_postanth*)**	102.05	**C**	120.39	**C**

*** indicate significant relationships (*p* < 0.001). Capital letters indicate significantly different mean values, comparingthem to LSD (least significant difference by Student’s *t*-test. DS—drought sensitive; DT—drought tolerant; HS—heat sensitive; HT—heat tolerant. *DS_anth*: drought stress at anthesis; *HT_anth*: high temperature at anthesis; *CMB_anth*: combined stress at anthesis; *DS_postanth*: drought stress at post-anthesis; *HT_postanth*: high temperature at post-anthesis; *CMB_postanth*: combined stress at post-anthesis.

## Data Availability

All related data are available within the manuscript and Supplementary Materials.

## Supplementary Materials

The following are available online at https://www.mdpi.com/article/10.3390/plants11010001/s1, Table S1: The alteration of physiological parameters caused by the different abiotic conditions; Table S2: The alteration of protein composition caused by the different abiotic conditions; Table S3: Phytotron chamber settings according to heat stress conditions.
